# Genetic testing and counseling for hypertrophic cardiomyopathy: An evidence‐based practice resource of the National Society of Genetic Counselors

**DOI:** 10.1002/jgc4.1993

**Published:** 2024-11-01

**Authors:** Erin M. Miller, Emily Brown, Susan Christian, Melissa A. Kelly, Linda M. Knight, Sara Saberi, Christina Rigelsky, Jodie Ingles

**Affiliations:** ^1^ Department of Pediatrics, College of Medicine University of Cincinnati Cincinnati Ohio USA; ^2^ Division of Cardiology Cincinnati Children's Hospital Medical Center Cincinnati Ohio USA; ^3^ Division of Cardiology Johns Hopkins University Baltimore Maryland USA; ^4^ Department of Medical Genetics University of Alberta Edmonton Alberta Canada; ^5^ Department of Genomic Health, Geisinger Danville Pennsylvania USA; ^6^ Children's Healthcare of Atlanta Cardiology Atlanta Georgia USA; ^7^ Cardiovascular Medicine University of Michigan Ann Arbor Michigan USA; ^8^ Center for Personalized Genetic Healthcare Cleveland Clinic Cleveland Ohio USA; ^9^ Genomics and Inherited Disease Program Garvan Institute of Medical Research, and UNSW Sydney Sydney New South Wales Australia; ^10^ School of Clinical Medicine, Faculty of Medicine and Health UNSW Sydney Sydney New South Wales Australia

**Keywords:** cascade testing, genetic counseling, genetic testing, genome sequencing, hypertrophic cardiomyopathy, variant classification

## Abstract

Hypertrophic cardiomyopathy (HCM) is a common hereditary condition affecting approximately 1 in 500 adults. It is characterized by marked clinical heterogeneity with individuals experiencing minimal to no symptoms, while others may have more severe outcomes including heart failure and sudden cardiac death. Genetic testing for HCM is increasingly available due to advances in DNA sequencing technologies and reduced costs. While a diagnosis of HCM is a well‐supported indication for genetic testing and genetic counseling, incorporation of genetic services into the clinical setting is often limited outside of expert centers. As genetic counseling and testing have become more accessible and convenient, optimal integration of genomic data into the clinical care of individuals with HCM should be instituted, including delivery via genetic counseling. Drawing on recommendations from recent disease guidelines and systematic evidence reviews, we highlight key recommendations for HCM genetic testing and counseling. This practice resource provides a comprehensive framework to guide healthcare providers in the process of genetic test selection, variant classification, and cascade testing for genetic evaluation of HCM.

## 1 INTRODUCTION

Hypertrophic cardiomyopathy (HCM) is a common hereditary condition affecting individuals of all ages with a prevalence of 1 in 500 adults (Arbelo et al., 2023; Maron et al., 1995; Ommen et al., 2020). The condition is characterized by left ventricular hypertrophy (LVH), which is often asymmetric, measuring 15 mm or greater (or z‐score > 2 in children) and occurring in the absence of another cardiac or systemic disease capable of causing the degree of hypertrophy observed (Arbelo et al., 2023; Ommen et al., 2020). A diagnosis of HCM is established through cardiac imaging with echocardiography remaining the primary modality. Common histopathologic features identified at time of surgical intervention (myectomy, cardiac transplant) or post‐mortem evaluation include myocyte disarray, myocyte hypertrophy, and interstitial fibrosis. The clinical symptomatology can range from asymptomatic LVH to chest pain, progressive heart failure symptoms, arrhythmias, and seldomly, sudden cardiac death.

Genetic counseling is a process supporting families to understand and adapt to the medical, psychological, and familial implications of the genetic contributions to disease (National Society of Genetic Counselors' Definition Task Force et al., 2006). This involves both education and provision of information, but also psychosocial support, and it is the combination of these skill sets that has shown to improve knowledge, recall, and patient empowerment; improve satisfaction with decision making; and reduce anxiety (Austin et al., 2014; Edwards et al., 2008; Ison et al., 2019; Michie et al., 1997). In this document, we assert that all genetic counseling be performed by appropriately qualified healthcare professionals, which in many countries would include trained genetic counselors (GCs) but also may include genetic nurses and clinical or medical geneticists with HCM expertise or cardiology providers with genetics expertise. These qualifications differ by country and healthcare setting.

Multiple expert consensus documents address genetic testing and counseling in HCM (Arbelo et al., 2023; Ommen et al., 2020; Wilde et al., 2022). An International Expert Consensus Statement published in 2022 recommends that genetic testing should only be performed with appropriate genetic counseling and emphasizes the value genetic counseling adds to variant interpretation and risk stratification (Wilde et al., 2022). Recognizing that HCM is often familial, the *2020 American Heart Association and American College of Cardiology Guideline for the Diagnosis and Treatment of Patients with Hypertrophic Cardiomyopathy* also addresses the importance of counseling regarding genetic etiologies and family risk stratification (Ommen et al., 2020). Most recently, the European Society of Cardiology (ESC) Guidelines for Management of Cardiomyopathies likewise recommends cardiac genetic counseling (Class I) for any patients with inherited cardiomyopathy regardless of whether genetic testing is being considered (Arbelo et al., 2023). Importantly, only the recent ESC guideline includes a GC in the development. Indeed, many clinical guidelines and expert consensus statements fall short of addressing important genetic counseling considerations related to HCM, such as genetic test and laboratory selection, variant classification, when to consider genomic sequencing, psychosocial counseling issues, and how to approach family risk stratification and targeted variant testing. Our practice resource aims to fill that gap for healthcare professionals including GCs providing care related to HCM.

Genetic testing for HCM is increasingly available due to advances in DNA sequencing technologies and reduced costs (Rehder et al., 2021). Genetic testing may be fully or partially covered by third‐party payors, and many clinical genetic testing laboratories in the United States offer cash pay options. Some laboratories will perform targeted variant testing in at‐risk family members at no cost, for a limited amount of time, when a causal (likely pathogenic or pathogenic) variant is identified in the family. Changes in sample requirements have also made genetic testing more convenient with home collection options via cheek swab in addition to the traditional blood sample. Similarly, access has improved, with a growing number of GCs with specific expertise in cardiovascular diseases now practicing (as of June 14, 2023, the National Society of Genetic Counselors Cardiovascular Special Interest Group (CV SIG) included 294 members, although not all provide clinical genetic counseling services) and greater options for patients to meet with GCs via telehealth (Green et al., 2023). In the United States and Canada, patients and providers can search for GCs by specialty, location, and mode of service delivery through the National Society of Genetic Counselor's “Find a Genetic Counselor” directory (https://findageneticcounselor.nsgc.org/). Integration of genetic counseling and testing in clinical care outside of the United States and Canada continues to evolve; however, access remains a challenge.

While a diagnosis of HCM is a well‐supported indication for genetic testing and genetic counseling, incorporation of genetic services into the clinical setting is often limited outside of expert centers. As genetic counseling and testing have become more accessible and convenient, optimal integration of genomic data into the clinical care of individuals with HCM should be instituted, including delivery via genetic counseling. Two systematic evidence reviews (SERs) and meta‐analyses evaluate the diagnostic validity and clinical utility of genetic testing and the uptake and utility of genetic testing and genetic counseling for HCM (Christian et al., 2022; Cirino et al., 2022). These SERs and consensus of this author group are used to provide evidence‐informed clinical practice resource for genetic testing and genetic counseling for HCM.

## 2 RECOMMENDATION 1

*Genetic testing should be offered to all individuals with a suspected or confirmed clinical diagnosis of HCM in the setting of appropriate genetic counseling*.

Rationale: Genetic testing can have an important impact on the management of patients with HCM. Managing patient expectations regarding the benefits and limitations of genetic testing is important.

### 2.1 Assessing clinical phenotype

An individual's clinical phenotype includes both their clinical cardiac diagnosis and family history. The clinical cardiac diagnosis of HCM or unexplained LVH should be confirmed by review of medical records and include the degree of hypertrophy by cardiac imaging, presence of myocardial scar by cardiac magnetic resonance (CMR) imaging, and degree of left ventricle outflow obstruction, if any. It is important to consider other conditions that cause hypertrophy including pressure overload due to long‐standing hypertension or aortic stenosis or storage infiltrative disorders. While HCM can be diagnosed in individuals of all ages, the differential diagnoses in childhood should include inborn errors of metabolism (i.e., Pompe disease), malformation syndromes (i.e., Noonan syndrome), and neuromuscular disorders (i.e., Friedreich ataxia). Knowledge of the cardiac testing and clinical diagnosis of HCM requires specific clinical expertise, often requiring multidisciplinary input from specialized centers. The National Society of Genetic Counselors (NSGC) CV SIG provides a number of clinical resources including a “Toolkit for Reading Cardiovascular Medical Records” that was updated in May 2024 and is available to members (https://www.nsgc.org/Members/Special‐Interest‐Groups‐SIGs/Cardiovascular‐Genetics‐SIG). Collaboration between genetic and cardiology specialists is important. A three‐ to four‐generation, cardiology‐focused family history should be elicited as part of the genetic testing process. Table 1 provides additional guidance on family history review specific to HCM. The family history can inform genetic test selection and pre‐test probability (i.e., the chance that an individual will have a positive genetic test result) as well as clinical interpretation of genetic testing results including the potential for other hereditable cardiac diseases and genocopies. Family history information also guides recommendations for cardiac screening and genetic testing in the family. While family history may be taken prior to genetic testing, it can also be informative to gather this information after completion of proband genetic testing. Both clinical diagnosis and family history should be considered when assessing the potential for an underlying genetic etiology. When initiating genetic testing for HCM, it remains most informative to test the most severely affected person in the family. This is to minimize the potential for a negative genetic test result in a relative who may have LVH related to other factors. In some situations, the most informative individual to test may be deceased and options for post‐mortem genetic testing should be explored.

**TABLE 1:** Approach and suggested questions to elicit a complete family history for HCM.

Family history characteristic	Additional questions	Reasoning for additional clarification of family history
“Healthy” first‐degree relative of proband	Do they have cardiac symptoms? Chest pain, shortness of breath, palpitations, racing heart? Have they seen a cardiologist? If yes, why? symptoms? when? What tests were performed? ECG, ECHO, MRI, monitor, stress test? What were the results?	Document screening status of first‐degree relatives Relatives often identify as healthy but have not been evaluated. Targeted questions about symptoms and past evaluations may improve recall Testing via MRI and monitor may indicate prior abnormal baseline testing or concerning symptoms
Relatives with HCM	What age was HCM diagnosed? How did the HCM come to medical attention? Incidental diagnosis, symptoms? Is there a history of arrhythmia? Cardiac arrest, ICD, ICD discharges, atrial fibrillation, ablation? Were stress ECHO, MRI performed? Obstruction or scar detected? How has the HCM been treated? Medication, myectomy, ablation? Did they have genetic testing? Other medical concerns such as learning problems, congenital heart defect, short stature, kidney disease, skeletal muscle disease?	Details help understand the severity and the variability in the family and direct family screening recommendations HCM may be first detected via routine ECG performed by PCP or presurgical ECG Assess risk for arrhythmia, sudden death based on family history Obtain records and assist with genetic testing if not performed Assess for syndromic causes of LVH that may need more targeted gene panel
Relatives with heart attack (HA)	What do you know about the HA? Circumstances: during exertion, at rest, in hospital, at home How was this treated? Surgery, stents, bypass, ICD, medications? Was there a prior history of heart attack/heart disease? Any risk factors for coronary heart disease? Smoking, hypertension, diabetes, obesity, high cholesterol, etc.	Many cardiac events may be reported by the family as a heart attack. Try to understand the nature of the heart event, as heart attack is commonly used as a term for many events, not just coronary heart disease‐mediated myocardial infarction (MI) Targeted questions may distinguish the type of event; MI—comorbidities, stent, CABG Heart failure—in hospital, declining health Cardiac arrest—sudden, more likely with exertion, ICD Aortic dissection—surgery May distinguish whether the condition is primary or acquired
Relatives with sudden cardiac death or sudden unexplained death	Do you know the circumstances of the death? At rest, exertional, witnesses, CPR, AED used? Did the individual survive? Were they hospitalized before death? Was there a history of arrhythmia? ICD, ablation, medications? Did the deceased have comorbidities? Was an autopsy performed? Negative? positive? Cardiomegaly? Genetic testing? How long ago was the death?	Exertional sudden deaths can be concerning for cardiac arrest, though events are also common at rest. AED use indicates that a shockable rhythm was identified If there is a prior cardiac history, records may be available The family may have a copy of the autopsy report. Autopsy reports may be public record depending on local laws. Obtain and review the report critically (Basso, C 2021, PMID: 33740097) Samples from the autopsy may be available for genetic testing
Relatives with heart failure (HF)	What was the reason for HF? Enlarged heart, dilated or hypertrophic, HCM/LVH, acquired? Did they have heart transplant? If yes, what age? Was any additional testing done on the heart? Prior history of myocardial infarction, coronary artery disease? Did they have risk factors—smoking, obesity, high cholesterol, lifestyle, etc.?	Determine the cause of the HF if possible. A burned out HCM heart may later convert to a HF/DCM phenotype Ischemic heart disease (myocardial infarction and coronary heart disease) is the most common cause for heart failure
Relatives with enlarged heart/thick heart	What part of the heart was enlarged? Dilated or thick? Did the cardiologist recommend family screening? Other reasons for thick heart? Competitive athlete, uncontrolled high blood pressure	Enlarged heart is a common term used for dilated cardiomyopathy and/or heart failure (see above) but could also be used for LVH. If the diagnosis is HCM, the cardiologist is likely to have recommended family screening
Relatives with valve disease	What valve was affected? Did they require surgery?	Aortic valve stenosis can cause LVH Mitral valve pathology can be seen in people with HCM; sometimes before LVH or obstructive disease is identified

*Note*: Eliciting an informative family history requires additional questions to understand the nature of the heart disease in the family. Family history knowledge is variable among family members but can impact medical recommendations. Targeted prompts and discussion can provide clarity and confirmation of reported family history and help to determine whether the condition is familial.

### 2.2 Pre‐test probability

**FIGURE 1:**
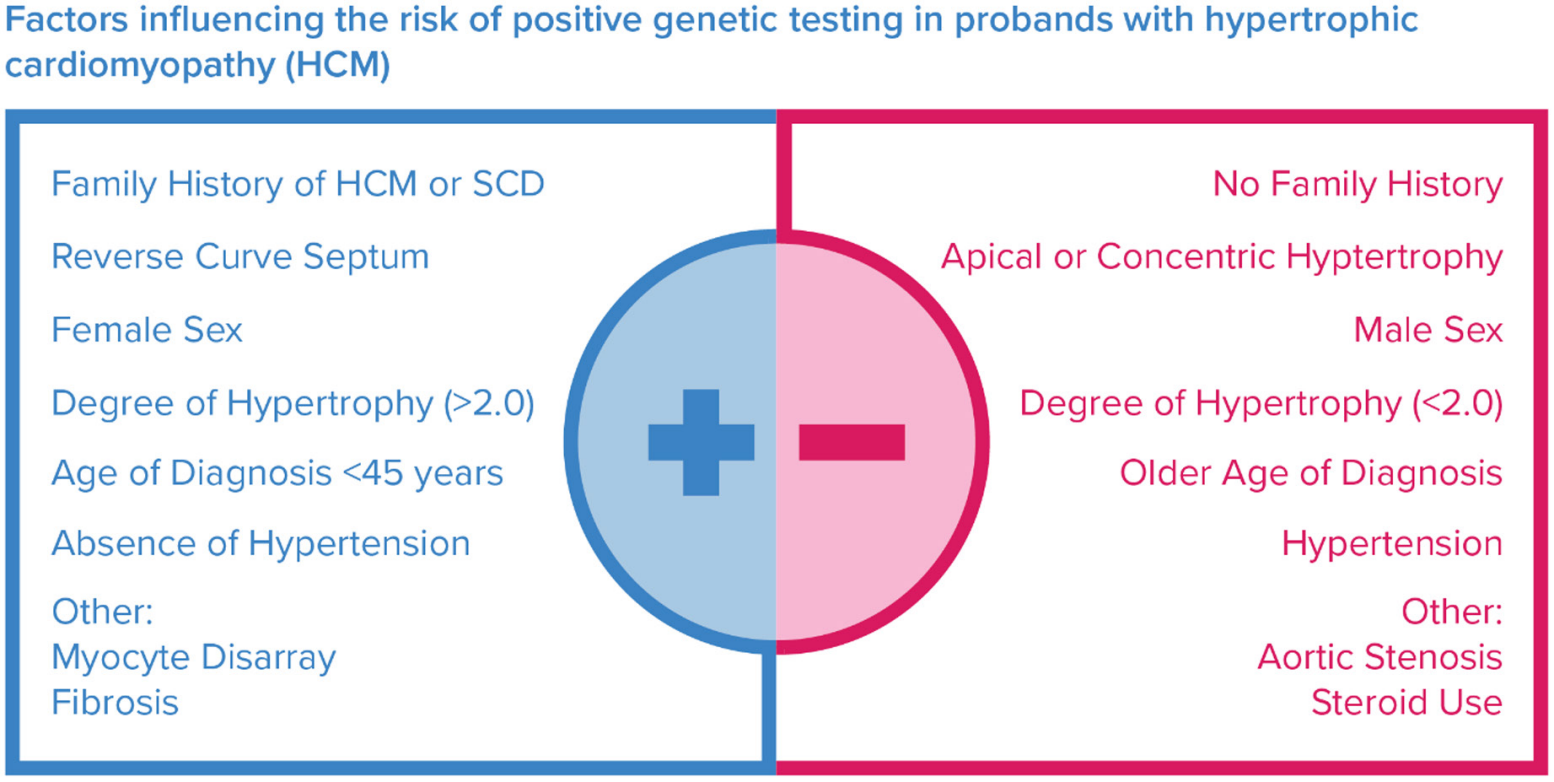
Factors influencing the risk of positive genetic testing in probands with hypertrophic cardiomyopathy (HCM).

Clinical information and family history inform the likelihood of a positive genetic test result. Establishing the pre‐test probability is intended to guide the clinical interpretation of genetic testing results and recommendations for additional genetic testing in the patient and cardiac screening in the family. Genetic testing prediction scoring systems incorporating clinical phenotype and family history are available for adults and children to assess pre‐test probability (Bos et al., 2014; Gruner et al., 2013; Ingles et al., 2017; Newman et al., 2018). Clinical and family history factors used to predict genetic testing results are summarized in Figure 1. Younger age at diagnosis increases the likelihood of a positive genetic testing result. The overall detection rate of genetic testing based on the presence of a disease‐causing variant among individuals ≤21 years is 78% compared to 33% among individuals diagnosed as an adult (Christian et al., 2022). These diagnostic yields are based on variant classification utilizing the current American College of Medical Genetics and Genomics and Association of Molecular Pathologists (ACMG/AMP) standards (Richards et al., 2015). In addition to younger age of onset, individuals assigned female at birth, individuals with a family history of HCM and/or sudden death, and individuals with a greater degree of asymmetric hypertrophy are more likely to have a positive genetic test result (Stafford et al., 2022). Lower pre‐test probability, however, should not preclude offering genetic testing. All individuals with a clinical diagnosis of HCM or unexplained LVH should be offered genetic testing regardless of age of onset, family history, or degree of hypertrophy. In certain clinical scenarios, LVH may be accompanied by other cardiac phenotypes including LV hypertrabeculation, heart failure, and myocarditis. The potential detiologies of the cardiac presentation should be discussed with the family, and genetic testing for HCM should be considered if the cause of LVH remains unclear.

### 2.3 Implications of genetic testing on management

Genetic test results can have important management implications as they enable identification of individuals with syndromic forms of LVH rather than non‐syndromic HCM most often caused by a variant in sarcomere protein genes. While patients with a syndromic form often present with multisystem involvement, this is not always the case. For example, individuals reported as male with an isolated cardiac variant causing Fabry disease may only present with LVH in the absence of extracardiac features (Nakao et al., 1995; Sachdev et al., 2002). Furthermore, LVH has been reported as the only sequelae for some X‐linked heterozygous individuals with a *GLA* variant associated with Fabry disease (Chimenti et al., 2004). Individuals with RASopathies can have highly variable phenotypes, and in some cases, LVH is the presenting feature, with less overt extra‐cardiac syndromic features that are not noted by the cardiologist (Brown & Murphy, 2021; Calcagni et al., 2018). Similarly, while Danon disease typically presents as a multisystem glycogen storage disorder, there are cases where the presentation is primarily LVH (often severe) that is misattributed to HCM (Arad et al., 2005).

Correctly identifying syndromic forms of LVH allows for correct medical therapies. For example, there are specific therapeutics for both Fabry disease and hereditary transthyretin amyloidosis (hATTR). In both conditions, it is critical to make the correct diagnosis given the medications treat the underlying disease mechanism and can significantly impact outcomes (Pieroni et al., 2022; Ruberg et al., 2019). Furthermore, individuals with amyloidosis do not respond well to beta‐blockers or ACE inhibitors, medications commonly prescribed for patients with HCM (Ruberg et al., 2019).

Additionally, because syndromic forms of LVH often involve other organ systems, correct diagnosis can lead to appropriate referrals, screening, and treatment beyond the cardiac phenotype. Importantly, Noonan syndrome is associated with an increased risk for hematologic malignancies and bleeding disorders, which need to be evaluated prior to surgeries (Romano et al., 2010). Referral to a neurologist is indicated for patients with transthyretin amyloidosis, Danon disease, Fabry disease, and mitochondrial disease to allow for appropriate management of the additional manifestations (Germain et al., 2019; Novelli et al., 2020; Ruberg et al., 2019).

Beyond identification of genetic syndromes, genetic testing can provide information regarding prognosis. A meta‐analysis (Christian et al., 2022) found that patients with positive genetic test results were more likely to undergo implantable cardioverter defibrillator (ICD) implantation, and evidence suggests that the presence of multiple disease‐causing variants is correlated with more severe disease (Burns, Bagnall, et al., 2017; van Velzen et al., 2018; Wang et al., 2014). There is limited evidence supporting further genotype–phenotype associations across multiple studies. However, data from the Sarcomeric Human Cardiomyopathy Registry (SHaRe) found that patients with sarcomere disease‐causing variants had a four times greater risk for transplant and two times greater risk for malignant arrhythmias, heart failure, and atrial fibrillation compared to patients with no identified disease‐causing variant (Ho et al., 2018). These results are echoed by other studies which found that individuals with non‐sarcomeric, non‐familial HCM had a less severe course of the disease and reduced risk of disease in at‐risk relatives (Curran et al., 2023; Ingles et al., 2017; Ko et al., 2018).

### 2.4 Psychosocial support needs

The psychological impact of having HCM is well documented. There can be difficulty adjusting to a new diagnosis, living with an ICD and the experiences in the family (Ingles, 2020). The impact of a young sudden cardiac death on the immediate relatives can be profound, including a high rate of distress, prolonged grief, and post‐traumatic stress (Ingles et al., 2016). There is an important need for referral to a clinical psychologist, and this is recommended in recent clinical guidelines (Stiles et al., 2021). There is no evidence to suggest that HCM genetic testing causes psychological distress (Ingles, 2020); however, when discussing HCM genetic testing with families, the psychological wellbeing of the patient should be considered. Counseling and psychological support resources may not be readily or widely available for individuals and families affected by HCM. Therefore, the genetic counseling should include short‐term client‐centered counseling which may include anticipatory guidance and employing crisis‐intervention skills such as active listening, empathy, and problem solving to create a plan for next steps and bridge to future interventions (Austin et al., 2014). Other resources that may be valuable include educational letters and materials to share with schools and employers, peer support groups, advocacy organizations, and opportunities for participation in research.

## 3 RECOMMENDATION 2

*Genetic tests should be selected, ordered, and interpreted in the setting of appropriate genetic counseling*.

**FIGURE 2:**
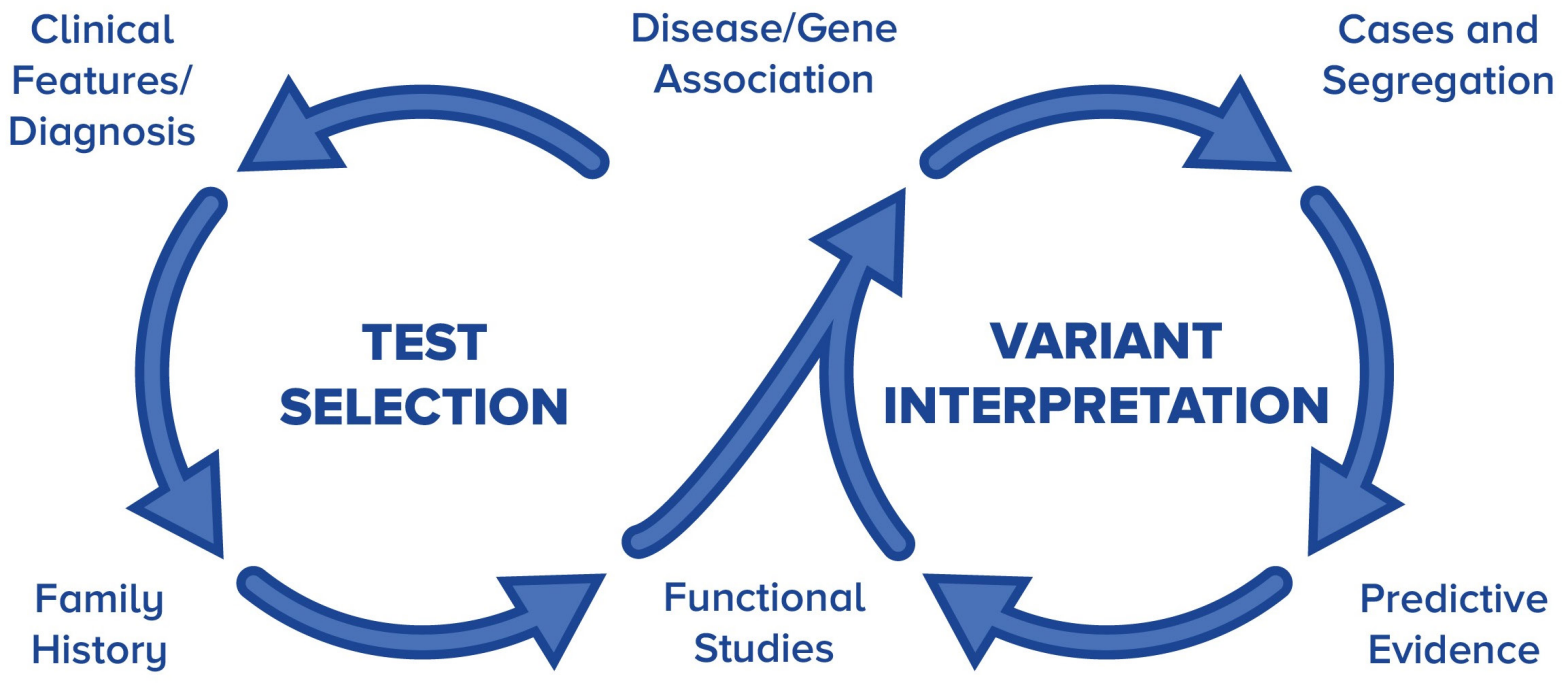
Components and considerations in a dynamic genetic testing process.

Rationale: Genetic testing can be a complex and dynamic process. When performing proband genetic testing, expertise is needed to identify the best genetic testing strategy; select the most appropriate test and reputable, certified laboratory; and provide clinical interpretation of the result based on the variant classification (Figure 2). If a causal variant is identified, cascade genetic testing of at‐risk relatives is indicated.

### 3.1 Test selection

A phenotype‐focused gene panel is recommended when a clinical diagnosis of HCM is confirmed based on the current understanding of diagnostic yield, with variants in sarcomeric genes (e.g., *MYBPC3*, *MYH7*, *TNNT2*, *TNNI3*, *TPM1*, *ACTC1*, *MYL2*, *TNNC1*, and *MYL3*) accounting for the vast majority of positive results (Alfares et al., 2015; Christian et al., 2022; Hespe et al., 2024; Ingles et al., 2019).

Among all gene positive cases, *MYBPC3* and *MYH7* account for 83%, and *TNNI3* and *TNNT2* account for 9% of causal variants. An increase in the panel size is not correlated with improved diagnostic testing yield. As a minimum, genetic testing should include genes with robust gene–disease association for non‐syndromic HCM and inclusion of genes associated with syndromic forms of LVH may be valuable in some instances. Table 2 includes key genes that should be considered. It is worth noting some apparent non‐syndromic HCM can in fact be due to syndromic genes, and in those instances, the extra‐cardiac features may not be recognizable by the clinical team. A mitochondrial gene, *MT‐TI*, has now been included in the list of genes with robust gene–disease association (Table 2). Mitochondrial gene sequencing can pose challenges for clinical laboratories to include in genetic testing; therefore, it is important that GCs can identify those patients where testing of this gene may be advantageous (i.e., likely monogenic, mitochondrial inheritance demonstrated on family history). Although multiple guidelines support genetic testing for HCM, the integration of genetic testing in clinical services remains center, provider, and country dependent (Ware et al., 2021).

**TABLE 2:** Genes with robust gene–disease association to be included in genetic testing for HCM from ClinGen HCM Re‐Appraisal (Adapted from Hespe et al., 2024).

Gene	Disease	Strength of evidence	Inheritance	Evidence‐associated phenotype
*ACTC1*	HCM	Definitive	Autosomal dominant	HCM
*ACTN2*	Intrinsic CM	Definitive	Autosomal dominant	LV cardiomyopathy (including hypertrophy, dilation, restrictive, hypertrabeculation/LVNC), arrhythmia
*ALPK3*	HCM	Strong	Autosomal dominant	HCM
*CACNA1C*	Timothy syndrome	Definitive	Autosomal dominant	LVH, prolonged QT interval, conduction disease, Timothy syndrome
*CSRP3*	HCM	Definitive	Semi‐dominant	HCM
*DES*	Desminopathy	Definitive	Autosomal dominant	LV cardiomyopathy (including hypertrophy, dilation, restrictive, hypertrabeculation/LVNC), arrhythmia, myofibrillar myopathy, neurogenic scapuloperoneal syndrome, Limb girdle muscular dystrophy
*FHL1*	Emery–Dreifuss MD	Definitive	X‐linked	LVH, conduction abnormalities, and Emery–Dreifuss MD
*FHOD3*	HCM	Definitive	Autosomal dominant	HCM
*FLNC*	Myofibrillar myopathy	Definitive	Autosomal dominant	LVH, RCM, and myofibrillar myopathy
*GLA*	Fabry disease	Definitive	X‐linked	LVH, Fabry disease
*JPH2*	HCM	Moderate	Autosomal dominant	HCM
*KLHL24*	HCM	Moderate	Autosomal recessive	HCM
*LAMP2*	Danon disease	Definitive	X‐linked	LVH, pre‐excitation syndromes, Danon disease
*MT‐TI*	HCM	Moderate	Mitochondrial	HCM
*MYBPC3*	HCM	Definitive	Autosomal dominant	HCM
*MYH7*	HCM	Definitive	Autosomal dominant	HCM
*MYL2*	HCM	Definitive	Autosomal dominant	HCM
*MYL3*	HCM	Definitive	Autosomal dominant	HCM
*PLN*	Intrinsic CM	Definitive	Autosomal dominant	LV or biventricular cardiomyopathy (including hypertrophy, dilation, restrictive, hypertrabeculation/LVNC)
*PRKAG2*	Cardiomyopathy	Definitive	Autosomal dominant	LVH and pre‐excitation syndromes
*PTPN11*	Noonan syndrome	Definitive	Autosomal dominant	LVH, septal defects, Noonan syndrome
*RAF1*	Noonan syndrome	Definitive	Autosomal dominant	LVH, septal defects, Noonan syndrome
*RIT1*	Noonan syndrome	Definitive	Autosomal dominant	LVH, septal defects, Noonan syndrome
*TNNC1*	HCM	Definitive	Autosomal dominant	HCM
*TNNI3*	HCM	Definitive	Autosomal dominant	HCM
*TNNT2*	HCM	Definitive	Autosomal dominant	HCM
*TPM1*	HCM	Definitive	Autosomal dominant	HCM
*TRIM63*	HCM	Moderate	Autosomal recessive	HCM
*TTR*	Transthyretin amyloidosis	Definitive	Autosomal dominant	LVH and amyloidosis

*Note*: All data and materials have been made publicly available on the ClinGen website: https://search.clinicalgenome.org/kb/affiliate/10104?page=1&size=25&search=.

Abbreviations: ACM, arrhythmogenic cardiomyopathy; ARVC, arrhythmogenic right ventricular cardiomyopathy; DCM, dilated cardiomyopathy; LOF, loss of function; LVNC, left ventricular non‐compaction; MD, muscular dystrophy; RCM, restrictive cardiomyopathy.

While ClinGen gene curation efforts have established a limited number of genes with the most robust gene–disease association that should be included in testing panels (Ingles et al., 2019), these are constantly evolving and being refined. Based on a recent ClinGen re‐appraisal of HCM genes, 29 genes are now considered to be HCM‐associated genes (Hespe et al., 2024). The most up‐to‐date list of HCM‐associated genes can be found on the ClinGen website (https://search.clinicalgenome.org/kb/affiliate/10104?page=1&size=25&search=).

In some scenarios, expanded genetic testing may be indicated. Special consideration of genetic testing approach is important when there are multiple cardiomyopathy phenotypes in a family, when the clinical cardiac phenotype is complex, if there are features suggestive of a genetic syndrome, or infantile‐onset for which sarcomere gene variants are less often identified (Stafford et al., 2022). Multi‐gene panel testing remains preferable in most, but not all, situations. Both exome and genome sequencing can identify sequence variants in genes known to be associated with HCM as well as variants in other protein coding genes (that may or may not be related to a diagnosis of HCM). In addition, genome sequencing can also identify non‐coding sequence variants outside of the exons that may affect gene activity and protein function. These deep intronic and other non‐coding sequence variants cannot be detected by gene panel or exome testing. Significantly, intronic variants in *MYBPC3* that affect splicing have been identified as an important cause of HCM, albeit a small contributor, highlighting the role of sequencing beyond coding regions (Bagnall et al., 2018). While exome and genome sequencing can identify variants not covered by phenotype‐focused gene panel testing, many of these variants may be of uncertain significance.

The diagnostic yield of genome sequencing in HCM has been compared to panel‐based testing and results in a similar yield (Christian et al., 2022; Cirino et al., 2022). Previously, variants that would be identified by panel‐based testing could sometimes be missed by exome or genome sequencing due to sub‐optimal coverage of certain genes; however, this is less of an issue with improving technology. While exome and genome sequencing are clinically available to individuals with HCM, there is variability in the cost and payer coverage for these tests. With multiple and emerging genetic testing options, resources like the Genetic Testing Registry can be helpful in understanding current testing options (https://www.ncbi.nlm.nih.gov/gtr/). Ultimately, exome and genome sequencing may be appropriate in specific scenarios for some individuals or families with HCM, not all individuals need expanded testing, and it should be considered on a case‐by‐case basis.

### 3.2 Laboratory selection

When selecting a laboratory to perform genetic testing, several key factors should be considered. It is important to consider the laboratory experience with HCM and cardiomyopathy genetic testing and variant classification. Cardiomyopathies are complex, heterogeneous diseases with multifaceted classification considerations. Laboratories that have been involved in the cardiovascular genetic testing space for the longest are most likely to have the greatest experience and appreciation for the nuances of these genes and variants. It is important to consider the composition of the testing panels offered and what technologies are used. The laboratory should include, at minimum, key genes with robust gene–disease association for both HCM and syndromic phenotypes that can include isolated LVH (Hespe et al., 2024; Ingles et al., 2019), but may vary in other genes included. There is often not a standard approach to the curation of gene panels by genetic testing laboratories in regard to which genes are included or the frequency of review and revision. The content of the specific gene panel should be reviewed before selecting a test and balanced with the individual's phenotype and family history. Additionally, the gene content of the panel may change over time and should be periodically evaluated.

Consideration of test methodology and the spectrum of variation detected, particularly larger deletions and duplications, is important for certain genes (e.g., *MYBPC3*). While most HCM genes can be adequately tested through next‐generation sequencing technologies, these may not provide comprehensive coverage for all relevant variant types in some cases and testing approach should be based on the phenotype or diagnosis of interest. An example of this is Friedreich's ataxia, an autosomal recessive trinucleotide repeat expansion disorder that can present with syndromic LVH, and typically requires different sequencing and/or analysis approaches for accurate detection.

The laboratory should meet all technical, regulatory, and certification requirements as designated per country or region to ensure the accuracy and reliability of the result. As cardiomyopathies and the underlying genetic etiologies are complex, we strongly encourage both provider and laboratory participation in knowledge sharing efforts (i.e., ClinGen) to collectively help advance the genomic understanding of this area (Rehm et al., 2015).

### 3.3 Determining the significance of a variant

#### 3.3.1 Variant classification

All variants identified through genetic testing should be classified by the laboratory based on the ACMG/AMP standards for variant classification (Richards et al., 2015). This framework is established for use in Mendelian disorders when disease–gene associations are known, however should not be used for classification of low penetrance variants or risk alleles. The framework focuses on key lines of evidence that include the variant type (i.e., missense, nonsense, frameshift), variant location, how often it is observed with disease versus in controls or healthy individuals, whether the variant has been reported to segregate with disease within a family, and published studies of the functional effect of the variant. Gene‐specific modifications have been developed for HCM (Kelly et al., 2018) and updated in the ClinGen Specifications Registry (https://cspec.genome.network/cspec/ui/svi/doc/GN002). It is important to note that despite ongoing efforts to improve variant classification, clinical genetic testing laboratories may still not align on classification of all variants, and it is important for ordering providers to understand the underlying variant evidence driving each laboratory's classification. Resources for variant classification are shown in Table 3.

**TABLE 3:** Resources for variant classification and clinical interpretation.

Resource	Website	Notes
Clinical Genome Resource (ClinGen)	https://clinicalgenome.org/	National Institutes of Health‐funded resource dedicated to building a central resource that defines the clinical relevance of genes and variants for use in precision medicine and research
NCBI ClinVar	https://www.ncbi.nlm.nih.gov/clinvar/	ClinVar is a freely accessible, public archive of reports of the relationships among human variations and phenotypes, with supporting evidence
Genome Aggregation Database (gnomAD)	https://gnomad.broadinstitute.org/	The Genome Aggregation Database (gnomAD) is a resource developed by an international coalition of investigators, with the goal of aggregating and harmonizing both exome and genome sequencing data from a wide variety of large‐scale sequencing projects and making summary data available for the wider scientific community
The Gene Curation Coalition (GenCC)	https://search.thegencc.org/	The GenCC DB provides information pertaining to the validity of gene–disease relationships, with a current focus on Mendelian diseases. Curated gene–disease relationships are submitted by GenCC member organizations
Varsome	https://varsome.com/	VarSome.com is a community‐driven project aimed at sharing global expertise on human variants. It features a robust aggregated knowledge base consisting of over 140 cross‐referenced data resources and contributions from its community of more than 500,000 users worldwide
DatabasE of genomiC varIation and Phenotype in Humans using Ensembl Resources (DECIPHER)	https://www.deciphergenomics.org/	DECIPHER is used by the clinical community to share and compare phenotypic and genotypic data. DECIPHER (DatabasE of genomiC varIation and Phenotype in Humans using Ensembl Resources) is an interactive web‐based database which incorporates a suite of tools designed to aid the interpretation of genomic variants
Cardiovascular Genomics and Precision Medicine Group (cardiodb)	https://www.cardiodb.org/	Collection of publicly available resources and applications based on published research studies from Cardiovascular Genomics and Precision Medicine Group, Imperial College London, including cardioclassifier, Atlas of Cardiac Variation, etc.
American Heart Association Genomic and Precision Medicine Council	https://www.ahajournals.org/circgen/bootcamp‐resources	Publicly available bootcamp resources developed by the Genomic and Precision Medicine Council of the American Heart Association
CardioGenomic Testing Alliance (CGTA)	https://cardiogenomictesting.com/	Comprised of a group of genomics companies and laboratories with the goal of raising awareness and improving utilization of genetic testing in cardiology

#### 3.3.2 Clinical interpretation

Clinical interpretation is the process of assessing variant pathogenicity in the context of the specific patient scenario. The clinical team should consider the laboratory classification, correlation of the gene to the clinical phenotype of the patient, and the family history and inheritance pattern. This clinical interpretation is necessary when testing via a comprehensive cardiomyopathy gene panel as panels may include genes with limited evidence. In addition, genetic testing reports may include multiple variants, and the clinical team must ultimately determine and assess whether any of the variants are a good candidate for the underlying cause of disease. With specific regard to variants of uncertain significance (VUSs), careful consideration is needed before acting clinically; a variant may have a strong clinical correlation with a phenotype and family history for a particular patient, but it does not automatically meet the same prediction thresholds for clinical action, and often, additional studies may be warranted to clarify causality.

Finally, in some instances where the clinical interpretation leads to increased suspicion about a particular variant, testing of additional relatives may be informative to help reject or support the pathogenicity of the variant. Family variant testing may be informative if a specific variant is found to be de novo or if the variant tracks with the cardiac phenotype in the family. Working with a team experienced in cardiomyopathy genetics, including cardiac GCs, is essential for genetic testing decisions especially in the context of a VUS.

#### 3.3.3 Incidental and secondary genetic findings

The identification of a genetic variant not related to the primary goal of the genetic testing is considered an incidental or secondary genetic finding. As genetic testing approaches become more comprehensive, incidental and secondary findings are increasingly common. The ACMG/AMP have developed a list of genes to guide the reporting of likely pathogenic and pathogenic variants considered secondary during diagnostic exome and genome testing (Miller et al., 2023). For patients with HCM where more comprehensive genetic testing is undertaken, there is risk of secondary genetic findings, including from identification of variants in other cardiac genes.

The ACMG/AMP secondary findings gene list includes definitive HCM sarcomere genes and a number of other genes with well‐established disease risk. Thus, providers may be asked to provide post‐test counseling related to secondary findings associated with HCM risk. It is important to consider that the presence or absence of a cardiomyopathy diagnosis cannot be established without cardiac imaging as many affected individuals may be asymptomatic. In addition, if an HCM‐associated variant is identified in an unaffected individual, with no family history of HCM, there may be lower disease penetrance (Bourfiss et al., 2022; McGurk et al., 2023; Topriceanu et al., 2024).

#### 3.3.4 Variant reclassification

Disease, gene, and variant‐level knowledge is constantly evolving, and as such, variant classifications may change over time. Discussion about the potential for variant reclassification is an important aspect of pre‐ and post‐test genetic counseling. However, the practicalities of ongoing, systematic variant reclassification are challenging. The need to reevaluate evidence over time raises issues regarding who is responsible for the ongoing review of variants: the laboratory, the clinician who ordered the test, or the family (Cherny et al., 2021; Das et al., 2014; Davies et al., 2021). Furthermore, a cadence of variant review has not been formally established, though it is generally suggested that this should occur every 1–3 years (Ommen et al., 2020). Variant review can be performed or requested when patients return to cardiology clinic for care and should be performed for variants identified before 2015, when the most recent standards and guidelines for the interpretation of sequence variants were published, and when new clinically relevant information becomes available in an individual or a family. As variants classified as uncertain are the most likely to change classification, they should also be re‐evaluated on a regular basis.

## 4 RECOMMENDATION 3

*Family screening, including cardiac screening and cascade genetic testing, as appropriate, should be offered to at‐risk relatives. Cascade genetic testing should be offered in the setting of appropriate genetic counseling without limitation of age*.

**FIGURE 3:**
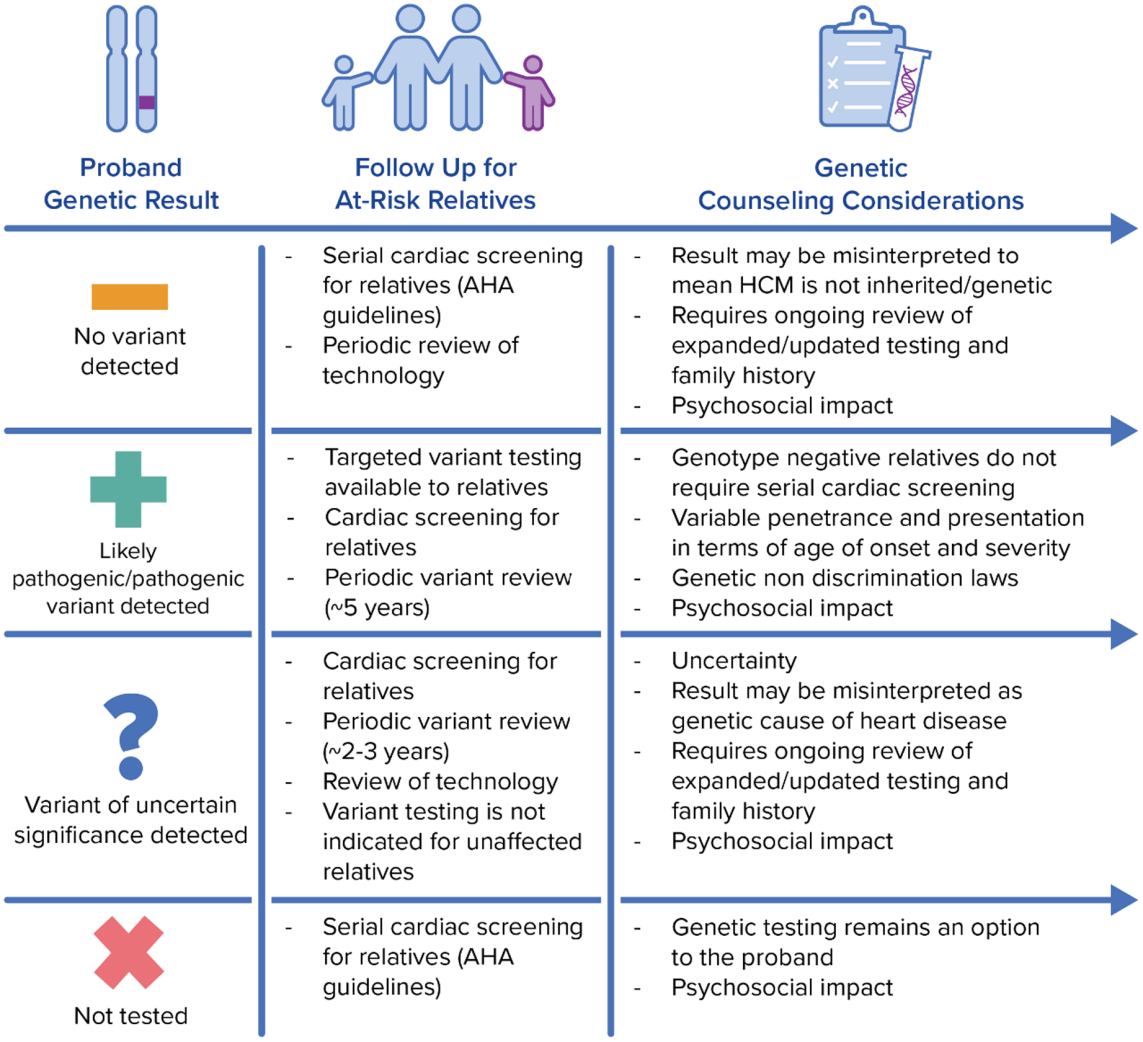
Family screening following proband genetic testing for HCM.

Rationale: Early diagnosis and management of HCM can improve long‐term cardiac outcomes (Arbelo et al., 2023; Ommen et al., 2020). Serial cardiac screening can allow clinical diagnosis in a pre‐symptomatic phase of disease, and when possible, testing for familial variants (cascade genetic testing) will further clarify which relatives require serial cardiac screening (Figure 3).

### 4.1 Family screening considerations

**FIGURE 4:**
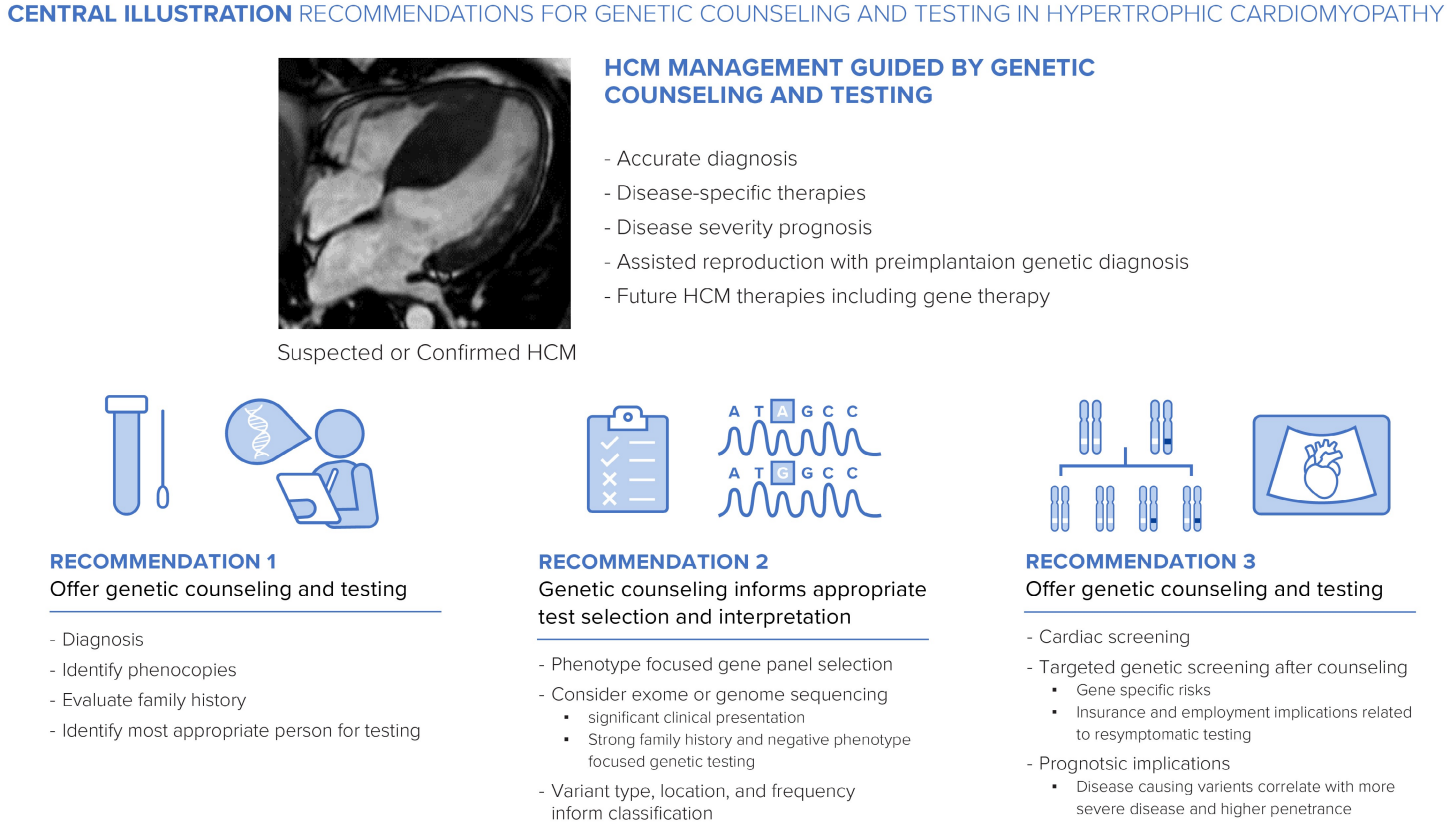
Central illustration recommendations for genetic counseling and testing in hypertrophic cardiomyopathy.

Family screening is the step‐wise, systematic process of monitoring relatives by cardiac screening, and as appropriate, cascade genetic testing for the causal variant in the family. Family screening is recommended for first‐degree relatives of patients with HCM regardless of age and proband genotype (Arbelo et al., 2023; Landstrom et al., 2021; Ommen et al., 2020). Despite recommendations for serial screening of at‐risk relatives, the average uptake is 69% and is significantly influenced by proband positive genotype (Cirino et al., 2022). Failure to identify a causal variant after genetic testing in the proband does not rule out a genetic cause. While there is increasing evidence to suggest that family members of gene negative probands may have lower risk and less frequent cardiac imaging may be appropriate, clinical guidelines so far err on a more conservative approach of continuing cardiac screening, although it is certainly an area for clinical judgment (Arbelo et al., 2023; Curran et al., 2023; Ommen et al., 2024). Due to limitations in the current methods of cardiac and genetic testing, the lack of empiric population data on recurrence risk in affected families, and a still evolving understanding of the pathogenesis of HCM, serial monitoring is recommended for first‐degree relatives in the absence of informative genetic testing in the proband (Figure 4).

### 4.2 Cardiac screening recommendations

Baseline cardiology testing is recommended for all first‐degree, at‐risk relatives and should include echocardiogram, electrocardiogram, and examination by a cardiologist (Arbelo et al., 2023; Hershberger et al., 2018; Ommen et al., 2020). The mean age of onset of HCM is in the third to fifth decade; however, a genotype‐positive relative may present infancy or never clinically manifest HCM (Girolami et al., 2023). A recent SER found an average penetrance of 62% across all studies and 24% during childhood (Christian et al., 2022). It is recommended that genotype‐positive relatives undergo serial cardiac screening by electrocardiogram and echocardiogram every 1–2 years through adolescence (<20 years) and every 3–5 years in adulthood (>21 years; Table 4) (Ommen et al., 2020). Cardiac screening intervals are more frequent in childhood, adolescence, and young adulthood (<25 years) or depending on family history. Indications to increase frequency may include young age of onset in the family, family history of severe disease and/or sudden cardiac death, and the presence of multiple genetic variants (Ommen et al., 2020). Conversely, there may be indications where less frequent cardiac screening can be considered for at‐risk family members. Factors that may indicate lower risk for monogenic disease in the family include older age of HCM diagnosis, presence of modifiable risk factors (i.e., obesity, hypertension), negative family history of HCM, and sudden cardiac death. When an at‐risk relatives present with cardiac symptoms, these should be immediately investigated. Clinical management, initiation of therapies, and lifestyle advice (such as physical exercise restrictions) are typically only indicated based on a clinical diagnosis of HCM, and not for genotype‐positive but clinically unaffected relatives. Cardiac screening considerations are covered by existing clinical guidelines and should be directed by the cardiologist (Arbelo et al., 2023; Ommen et al., 2020).

**TABLE 4:** American Heart Association/American College of Cardiology HCM family screening guidelines.

AHA/ACC hypertrophic cardiomyopathy family cardiology screening guidelines		
Serial Screening to include ECG and echocardiogram		
Proband Gene Status	Children 0–20 years	Adults >20 years*
Genotype positive Genotype unknown Genotype VUS (compelling) Early‐onset family history	At time of proband diagnosis regardless of age of onset in the affected relative Repeat every 1–2 years	Every 3–5 years
Genotype negative	At any age regardless of the age of onset in the affected relative, but no later than puberty Repeat every 2–3 years	Every 3–5 years *No recommended age at which screening can be discontinued

*Note*: Adapted from the American Heart Association/American College of Cardiology 2020 HCM Consensus Guidelines PMID: 33229116. All first‐degree relatives should have baseline cardiology screening. Relatives of probands with no genetic testing or a VUS and those with family history of early‐onset disease should be seen for routine and periodic screening. Relatives of genotype negative probands may delay and space out the screening interval.

Abbreviations: ECG, electrocardiogram; HCM, hypertrophic cardiomyopathy; VUS, variant of uncertain significance.

Family history of HCM is an item on the American Academy of Pediatrics recommended survey for pediatric sports participation clearance (Erickson et al., 2021) which does not distinguish the degree of relationship. Genetic counseling is recommended for these families.

### 4.3 Cascade genetic testing

Where a causal genetic variant is identified in a proband, targeted variant testing (cascade genetic testing) of first‐degree at‐risk relatives becomes available. Importantly, first‐degree at‐risk relatives have a 50% chance of inheriting the genetic variant, and discussion to weigh up the risks and benefits of cascade genetic testing should be offered. The key value of genetic testing for HCM arises from the opportunity to clarify genetic risk for relatives, as those that test negative for the familial variant may be released from serial cardiac screening and cannot pass HCM onto their biological children, whereas relatives who test positive for the familial variant are at increased risk of developing HCM in their lifetime and importantly means their biological children have a 50% chance of inheriting the genetic variant. Being able to cease serial cardiac screening in ~50% of at‐risk relatives is what drives HCM genetic testing being cost‐effective compared to clinical screening alone (Ingles et al., 2012; Wordsworth et al., 2010).

Pre‐test genetic counseling for relatives considering cascade genetic testing should include a discussion of incomplete penetrance and variable expression with regard to age of onset and disease severity. It is important to include a discussion of genetic discrimination, highlighting some of the limitations of genetic non‐discrimination laws in the United States; particularly that health insurance has protections but life, disability, and long‐term care insurance do not (Prince et al., 2022). Potential for genetic discrimination and insurance implications should be discussed according to the local context, noting that each country has unique concerns.

### 4.4 Cascade genetic testing in biological children

When a disease‐causing variant is identified for a parent with young children (<10 years), the family should be supported to determine the ideal timing of targeted familial variant testing for their at‐risk children. The majority of parents report that cascade genetic testing should be offered to them when their children are young, as an option to help clarify who requires ongoing cardiac screening (Christian et al., 2022). For families that choose to defer cascade genetic testing until children are older, serial cardiac screening is recommended as HCM has been reported across the lifespan (Kaski et al., 2009).

Decision aids are available and useful to assist families in deciding between serial cardiac screening and cascade genetic testing (Christian et al., 2021). While numerous associations and societies agree cascade genetic testing of minors is indicated (Ross et al., 2013) for monitoring of potential childhood onset of HCM, some centers recommend deferring testing until the child is of the age of assent (>10 years) (Girolami et al., 2023). If families choose cascade genetic testing for their young children, the children's concerns should be addressed as appropriate and assent obtained (Arbelo et al., 2023).

### 4.5 Reproductive genetic testing and planning

A parental diagnosis of HCM is an important consideration for family planning. A pre‐existing diagnosis of HCM can impact maternal health with potential risk for heart failure, stroke, and arrhythmia during pregnancy. Pregnant individuals with HCM should receive specialized pregnancy care including discussion of current cardiac medications (Sliwa et al., 2021). Regarding risk for HCM in future biological children, expectant couples should be offered pre‐conception genetic counseling. Reproductive genetic testing is increasingly available, though its availability depends on the country and health service (Verdonschot et al., 2024). Topics that can be addressed include the option for preimplantation genetic testing if a familial variant has been identified. In addition, options for fetal echocardiography and postnatal cardiac imaging should be discussed. This is an opportunity for collaboration across genetic counseling specialties including reproductive, prenatal, and cardiovascular to provide the best patient and family care.

### 4.6 Recommended follow‐up for families with a VUS

Cascade genetic testing of at‐risk relatives for a VUS is not recommended as the presence or absence of the VUS does not provide informative or actionable information. These relatives need serial cardiology monitoring. Variant segregation testing of informative relatives, such as parents or other affected relatives, for the purposes of clarifying whether the variant is causal can be helpful. Testing other affected relatives for the presence of a VUS may show co‐segregation with the condition and lend support to the pathogenicity of the variant or reveal non‐segregation providing evidence that the VUS is less likely the cause of disease in the family. Negative parental testing may prove de novo occurrence and support pathogenicity. In addition, testing elder relatives with normal cardiac screening for the presence of a VUS could refute pathogenicity, although non‐penetrance must be considered which limits utility. Periodic review of family history is important and may help to inform variant classification. As relatives pursue cardiac screening, their diagnostic status may change, which may impact recommendations for family variant testing. As family members may access genetic and cardiology care via different laboratories and care facilities, the clinical care team should collaborate closely with laboratory colleagues to improve segregation analysis.

### 4.7 Recommendations for relatives of a proband with an unknown genotype

While it is recommended that HCM panel testing be performed on the affected family member, for many reasons, this is not always possible (some examples may include strained family relations, deceased affected relatives, and the proband declining genetic testing). Panel‐based genetic testing (HCM panel or cardiomyopathy panel) is not recommended for at‐risk individuals without an affected family member who has been tested (Arbelo et al., 2023; Ommen et al., 2020). A negative result in this setting does not eliminate the risk of developing the condition. HCM panel testing yields at least one VUS in about 24% of patients (Christian et al., 2022). A VUS may be misinterpreted in the absence of a phenotype or an affected relative to test against a variant (Amin & Wilde, 2018; Burns, Yeates, et al., 2017). This may lead to a spurious, increased risk assessment and unnecessary patient distress. Based on current expert consensus statements, the recommendation for serial cardiology testing does not differ for at‐risk relatives based on a positive, negative, or inconclusive HCM gene panel test results without an informative genetic result in the proband.

### 4.8 Self‐directed genetic testing

Self‐directed genetic testing for heart disease is offered by clinical laboratories and available to consumers, including at‐risk relatives. Self‐directed genetic testing, also referred to as direct‐to‐consumer, refers to tests that are made accessible directly to customers and can be purchased directly from testing laboratories without involvement of a healthcare provider. Regarding self‐directed gene panels, these typically include genes for many types of inherited heart conditions and may include genes with limited evidence. Reporting protocols vary across laboratories; some may limit results to only disease‐causing variants, whereas others may report all variants or provide unclassified raw variant data. Many patients and families with HCM harbor DNA variants that are novel and classified as uncertain. Thus, a negative self‐directed panel that only reports out disease‐causing variants may provide false reassurance that the individual is not at risk for HCM. Third‐party interpretation services can also be accessed for many types of self‐directed genetic testing. Serial cardiac screening is still recommended for at‐risk relatives, and the interval does not differ based on a negative or inconclusive cardiac gene panel test results. Genetic counseling for self‐directed testing is discussed in a 2023 practice resource from the NSGC (Blout Zawatsky et al., 2023).

### 4.9 Family communication

Probands should be appropriately encouraged to share their HCM diagnosis and genetic test results with family members. A recent systematic review found that genetic counseling increased awareness of family screening recommendations, although genetic test results are not a predictor of family communication (Cirino et al., 2022). Various tools have been developed to assist with family communication for a number of inherited heart conditions including a family letter (van der Roest et al., 2009), communication aid (Burns et al., 2023), patient videos (Harris et al., 2019), and chat bot (Schmidlen et al., 2022). In addition, some centers have requested permission from the proband to contact their family members directly to discuss the risk of HCM in the family (van den Heuvel et al., 2022).

Many families have incomplete uptake of cascade genetic testing, leaving more distantly related family members uncertain about their risk status. These relatives can have an elevated risk of HCM compared to the general population. Cascade genetic testing of more distant relatives is indicated when intervening relatives have not been tested. Families need to be advised that a positive genetic testing result in this setting will reveal genotype status of the intervening but untested relatives. Additionally, when the genetic status of the proband is unknown or negative, serial cardiac screening is still appropriate. Families should be informed that like other heart conditions, many people with cardiomyopathy can be pre‐symptomatic and unaware that they have the condition (i.e., being asymptomatic is not equivalent to being unaffected). Limitations of a cardiac evaluation in predicting future health should be discussed along with the need for at‐risk relatives to pay attention to symptoms which may include irregular heart rate, dizziness, or syncope with exertion and seek cardiac screening at the recommended interval.

## 5 IMPLEMENTATION OF RECOMMENDATIONS

Research to inform how genetic testing and counseling should be implemented in the clinical setting is an important consideration. While we increasingly build our understanding of the underlying genetic drivers of disease, ensuring families can equitably access these services with appropriate genetic counseling is critical. Four key areas are described below as potential future areas of development that could impact HCM genetic counseling and testing.

### 5.1 Access to genetic counseling and genetic testing

Given the utility of genetic counseling, ensuring access to this service is key. Historically, access has been limited by a patient's location (Raspa et al., 2021). However, telehealth has greatly increased since the COVID‐19 pandemic (Weiner et al., 2021), and genetic counseling telehealth appointments are now offered by both independent companies and many hospitals and clinics (Shur et al., 2021). A SER found that telehealth genetic counseling appointments are non‐inferior and comparable to in‐person visits (Danylchuk et al., 2021). Alternative service delivery models that do not involve a GC may be appropriate to improve access to genetic testing, when access to a GC remains limited. Genetic counseling can be provided by a healthcare provider not trained as a GC but with expertise in cardiology and genetics.

### 5.2 Polygenic risk scores (PRSs)

It is now widely recognized that not all HCM is caused by monogenic variants in disease genes. Further, the marked clinical heterogeneity observed even among individuals with the same genotype highlights that clinical phenotype is not fully explained by monogenic variants. Genome‐wide association studies have demonstrated that genetic heritability can be further explained by the cumulative effects of common genetic variants (Harper et al., 2021; Tadros et al., 2023). It is likely that an integrated risk including PRS, other factors (e.g., blood pressure, sex), and presence of monogenic variants in disease‐associated genes will provide greater clarity around risk of disease and clinical outcomes, though research to guide clinical validation and implementation have yet to be undertaken. From a genetic counseling perspective, learning from other disease settings that have developed expertise and resources to convey both monogenic and polygenic disease risk and genetic results will be critical. A NSGC practice resource addressing PRSs provides greater in‐depth discussion and context and serves as a resource for GCs (Wand et al., 2023).

### 5.3 New and emerging therapies

An approaching wave of rare disease therapeutics that take advantage of new and emerging tools such as RNA therapeutics and CRISPR‐based technologies will likely impact the field of inherited cardiomyopathies in the future. Indeed, preclinical studies have shown the ability to correct the *MYH7* p.R403Q pathogenic variant in cardiomyocyte cell lines (Reichart et al., 2023). At present, Mavacamten, a novel allosteric inhibitor of myosin‐specific ATPase, is the only targeted therapy approved for use in patients with HCM (Spertus et al., 2021). Therapies that require genotype to be established as part of their eligibility for use may improve integration of genetic testing into routine cardiology care. There are multiple clinical trials investigating therapies for HCM, and patients and families should be informed of these opportunities (clinicaltrials.gov).

### 5.4 Toward health equity

Our historic focus on individuals of European ancestry in both research and genomic reference databases has created a disparity in outcomes from genetic testing across all disease settings, including HCM. Patients from poorly represented ancestry groups are 2–3 times more likely to receive an uncertain genetic result, limiting the benefit from a genetic diagnosis and increasing the risk of variant misclassification (Butters et al., 2021; Manrai et al., 2016). The value of studying diverse HCM populations globally has been shown numerous times, with increasing recognition of the need for diverse research teams (Allouba et al., 2023; Ingles & MacArthur, 2023). Likewise, race has been shown to be an important factor in overall clinical outcomes (Eberly et al., 2020), and indeed, it is recognized that the majority of clinical research that has formed the basis of our understanding of HCM has originated from only a small number of countries primarily of European ancestry. Efforts to increase diversity in research and reference databases have been recognized and are critical in ensuring that new discoveries in genomic medicine benefit the population equitably.

## 6 CONCLUSIONS

HCM is a heterogeneous genetic disease that has been the focus of intense research efforts for many decades. Genetic testing and counseling add value to management of patients with HCM and their at‐risk family members. Genetic counseling is critical, not just for enabling genetic testing but for educating patients, communicating inheritance risks, and psychosocial support. Genetic testing can improve diagnosis, clarify risk for family members, and allow reproductive options. While there are still gaps in knowledge that contribute to uncertainty and challenges in the field of HCM, GCs play an important role in patient management, genetic testing, education and research.

## 7 RESOURCES

Hypertrophic Cardiomyopathy Association: 4HCM.org.

Children's Cardiomyopathy Foundation, CCF:childrenscardiomyopathy.org.

A video describing genetic testing and family screening for cardiomyopathy can be found on YOUTUBE: https://www.youtube.com/watch?v=amxliwiaX0E.

Cardio What? A kids' guide to cardiomyopathy—a patient‐oriented pamphlet produced by the NSGC and CCF. Order at NSGC.org.

## AUTHOR CONTRIBUTIONS

All the authors gave final approval of this version to be published and agree to be accountable for all aspects of the work in ensuring that questions related to the accuracy or integrity of any part of the work are appropriately investigated and resolved.

## CONFLICT OF INTEREST STATEMENT

NSGC requires practice resource authors to complete a COI disclosure survey annually, starting at the formation of the author group. Authors must also report interim COI changes to the NSGC Practice Guideline Committee (PGC) within 30 days. The PGC categorizes COI into two tiers. Tier 1 COI includes any direct, personal financial benefit that is ongoing or within the previous 12 months from a commercial entity that may benefit from the document. Tier 1 COI includes research funding from a commercial entity for 25 percent or greater of an author's salary. Tier 2 COI includes limited consultant roles, paid stipends/travel, and ongoing consultancy roles with companies that are involved in healthcare, but may not directly benefit from the document. The PGC assesses the overall balance of COI for the author group and requires that no more than 40 percent of authors have Tier 1 COI and no more than 80 percent have either Tier 1 or Tier 2 COI. Lead authors must be free of Tier 1 COI for the entirety of the development of the document and can only have Tier 2 COI if serving alongside a co‐lead author with no Tier 1 or Tier 2 COI. Erin M. Miller, Emily Brown, Susan Christian, Melissa A. Kelly, Linda M. Knight, Sara Saberi, and Christina Rigelsky declare that they have no conflict of interest. Jodie Ingles receives research grant support from Bristol Myers Squibb.

## DISCLAIMER

This practice resource (PR) is provided by the NSGC solely to serve as a helpful practice management resource and tool for GCs and other healthcare providers. NSGC's PRs are not evidence‐based; instead, they are based on the personal recommendations and experience of the authors. Each NSGC PR focuses on a clinical or practice‐based issue, includes points for the GC or other healthcare provider to consider, and is based on the author(s) review and analysis of current professional literature believed to be reliable. As such, the information provided and ideas discussed in NSGC's PRs (a) reflect only the current scientific and clinical knowledge at the time of publication; (b) are only current as of their publication date; and (c) are subject to change without notice as advances emerge. PRs do not (and are not intended to) dictate an exclusive course of management nor guarantee a particular outcome. NSGC's PRs are never intended to displace a GC or other healthcare provider's best medical judgment based on the clinical circumstances of a particular patient or patient population. NSGC publishes PRs for educational and informational purposes only and does not “approve” or “endorse” any specific methods, practices, or sources of information contained therein.
